# Seroprevalence and Risk Factors of *Anaplasma* spp. in German Small Ruminant Flocks

**DOI:** 10.3390/ani11102793

**Published:** 2021-09-25

**Authors:** Wiebke Rubel, Clara Schoneberg, Annika Wolf, Martin Ganter, Benjamin Ulrich Bauer

**Affiliations:** 1 Clinic for Swine and Small Ruminants, Forensic Medicine and Ambulatory Service, University of Veterinary Medicine Hannover, Foundation, 30173 Hannover, Germany; Annika.Wolf@tiho-hannover.de (A.W.); Martin.Ganter@tiho-hannover.de (M.G.); Benjamin.Bauer@tiho-hannover.de (B.U.B.); 2 Department of Biometry, Epidemiology and Information Processing, WHO Collaborating Centre for Research and Training for Health at the Human-Animal-Environment Interface, University of Veterinary Medicine Hannover, Foundation, 30559 Hannover, Germany; Clara.Schoneberg@tiho-hannover.de

**Keywords:** *Anaplasma phagocytophilum*, *Anaplasma ovis*, tick-borne fever, ovine anaplasmosis, sheep, goat, risk factors, landscape conservation

## Abstract

**Simple Summary:**

*Anaplasma phagocytophilum* and *Anaplasma ovis*, tick-borne pathogens with zoonotic potential, have been detected in small ruminants in Europe and North America in the past. These intracellular bacteria cause tick borne fever and ovine anaplasmosis, respectively. The most common clinical signs of infection are fever, lethargy and anaemia. To date, little is known about the distribution of these pathogens in sheep and goats from Germany. Therefore, 3178 serum samples of small ruminants from 71 farms distributed in five German federal states (Schleswig-Holstein, Lower Saxony, North Rhine-Westphalia, Baden-Wuerttemberg and Bavaria) were examined for IgG antibodies to *Anaplasma* species by a cELISA based on the MSP5 antigen. In 70 flocks, antibodies to *Anaplasma* spp. were detected in both sheep and goats. Furthermore, a risk factor analysis was carried out by means of a questionnaire answered by the farmers. Older animals and females were more likely to have antibodies to *Anaplasma* spp. Moreover, sheep had a higher probability of becoming seropositive than goats. Using flocks for landscape conservation and the presence of cats and dogs on the farm increased the risk of having more than 20% seropositive animals within the flock significantly. Since antibodies to *Anaplasma* spp. have been detected in almost all flocks (70/71), it can be assumed that *Anaplasma* spp. might be underdiagnosed in small ruminants from Germany.

**Abstract:**

Knowledge about the distribution of *Anaplasma* spp. in small ruminants from Germany is limited. Therefore, serum samples were examined from 71 small ruminant flocks (2731 sheep, 447 goats) located in the five German federal states: Schleswig-Holstein (SH), Lower Saxony (LS), North Rhine-Westphalia (NRW), Baden-Wuerttemberg (BW) and Bavaria (BAV). Antibodies to *Anaplasma* spp. were determined by a cELISA based on the MSP5 antigen. A risk factor analysis at animal and flock level was also performed. Antibodies to *Anaplasma* spp. were detected in 70/71 flocks without significant difference in the intra-flock prevalence (IFP) between the federal states. The mean antibody levels from sheep were significantly lower in northern Germany (LS, SH) compared to west (NRW) and south Germany (BW, BAV). Sheep had a 2.5-fold higher risk of being seropositive than goats. Females and older animals (>2 years) were more likely to have antibodies to *Anaplasma* spp. in one third and one quarter of cases, respectively. Flocks used for landscape conservation had a five times higher risk of acquiring an IFP greater than 20%. Cats and dogs on the farms increased the probability for small ruminant flocks to have an IFP of above 20% 10-fold and 166-fold, respectively. Further studies are necessary to assess the impact of *Anaplasma* species on the health of small ruminants in Germany.

## 1. Introduction

Across Europe and North America, sheep and goats can become infected with obligate intracellular bacteria of the genus *Anaplasma*. Whereas *Anaplasma phagocytophilum* is widespread in many European countries, an infection with *Anaplasma ovis* mainly occurs in the Mediterranean Basin [1]. However, reports about the focal occurrence of *A. ovis* in Central European countries like Hungary, Slovakia and Germany are increasing [2,3,4]. Both pathogens are also present in the US sheep population, but detailed information about the dissemination is lacking [5,6].

Wild ruminants may act as a reservoir for both pathogens in Europe and North America [3,7,8,9,10,11]. The transmission of *Anaplasma* spp. usually happens through tick bites [1]. The main vectors of *A. phagocytophilum* are *Ixodes ricinus* in Europe, as well as *Ixodes scapularis*, *Ixodes pacificus* and *Ixodes spinipalpis* in North America [1,11,12,13]. Different tick species belonging to the genera *Dermacentor*, *Rhipicephalus* and *Hyalomma* are considered to transmit *A. ovis* [13,14]. In recent years, *A. ovis* was also found in sheep keds (*Melophagus ovinus*) but their vector competence remains doubtful [15,16].

The replication of *A. phagocytophilum* takes place within the vacuoles of neutrophil granulocytes and sometimes also lymphocytes [17]. This causes granulocytic anaplasmosis in many domestic animals, such as horses [18,19], cattle [20,21], dogs [22,23] and cats [24,25], and also in humans [12]. In small ruminants, *A. phagocytophilum* results in tick-borne fever (TBF) and affected animals suffer from high fever, anorexia and dullness [26,27,28]. Neutropenia and thrombocytopenia are the haematological key findings in affected sheep and goats [26,29]. Immunosuppression causes a high susceptibility to secondary infections like *Mannheima haemolytica* and *Bibersteina trehalosi* and leads to respiratory distress in lambs [30,31]. Furthermore, *A. phagocytophilum* favours co-infections with staphylococcal bacteria which cause tick-pyaemia with polyarthritis [29]. TBF and co-infections can be fatal for lambs [29,30,31]. However, mild courses of *A. phagocytophilum* were reported but affected lambs had reduced growth rates [28]. Goats show similar clinical signs to sheep after an infection with *A. phagocytophilum*, but to a lesser extent [26,32,33].

*Anaplasma ovis* mainly affects the ovine and caprine erythrocytes [34] but can also be found in wild ungulates like roe deer (*Capreolus capreolus*) and red deer (*Cervus elaphus*) [7,15,35]. Humans rarely become infected [36]. The pathogen causes ovine anaplasmosis especially in sheep in poor health [37]. Main clinical signs in sheep are fever, severe anaemia, extreme weakness, anorexia, and weight loss [34,37,38,39]. Moreover, haemoglobinuria and icteric carcasses were also described in sheep infected with *A. ovis* [2,39]. An acute infection results in decreased values of red blood cells, haemoglobin and packed cell volume [40]. Although the same signs are described for goats as for sheep, *A. ovis* appears to be more pathogenic for goats [41].

In Germany, *A. phagocytophilum* was identified in *I. ricinius* across the country with detection rates between 1.9% and 5.4% [42,43,44]. Although *A. phagocytophilum* has been well described in domestic animals [19,20,25,45] and wild ungulates [9,46], knowledge of the occurrence of the pathogen in German sheep and goat flocks is still limited. A molecular investigation revealed an infection rate of 4% (*n* = 255) in sheep from Northern Germany [8] and a clinical case of TBF was described in a goat from western Germany [47]. Recently, *A. phagocytophilum* was detected by PCR in five sheep flocks located in the southern part of the country [4]. In the same study, *A. ovis* was identified for the first time in a German sheep flock.

Due to the lack of information about the occurrence of *Anaplasma* spp. in the German small ruminant population, the present study aimed to determine the seroprevalence of *Anaplasma* spp. in sheep and goat flocks across Germany by using a cELISA to receive further information about the dissemination of *Anaplasma* species. Moreover, a risk factor analysis was performed to identify potential threats for sheep and goats in Germany to be exposed to *Anaplasma* species. This risk factor analysis was based on data from a standardised questionnaire which was performed with the sheep farmers [48].

## 2. Materials and Methods

### 2.1. Animals

**Figure 1:**
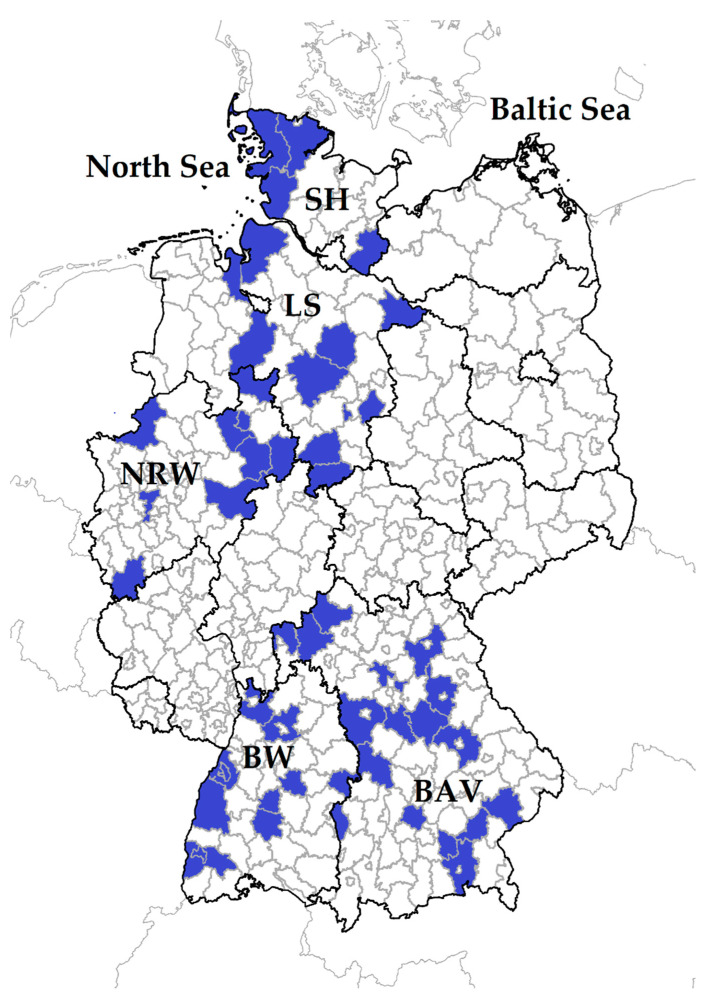
In total, 71 small ruminant flocks were sampled in five German federal states: Schleswig-Holstein: SH; Lower Saxony: LS, North Rhine-Westphalia: NRW, Baden-Wuerttemberg: BW, Bavaria: BAV. Districts with participating farms are coloured in blue.

Serum samples from sheep and goats were available from a Q fever study conducted from winter 2017 to spring 2018, and details were described elsewhere [49]. In brief, the specimens were collected from 3178 small ruminants (2731 sheep, 447 goats) within 71 flocks located in five federal states: Schleswig-Holstein (SH), Lower Saxony (LS), North Rhine-Westphalia (NRW), Baden-Wuerttemberg (BW) and Bavaria (BAV) (Figure 1). 

These states have the largest sheep populations within Germany and the farms were selected based on the owners’ willingness to participate in the study. The number of required samples to determine the intra-flock prevalence (IFP) was calculated on the assumption of 3% expected prevalence, 95% confidence interval, 80% power and 5% precision. A maximum of 44 animals per flock were sampled. In sheep flocks with goats, the sample size for the goats was calculated under the same assumptions as for sheep. Individual ear tag number, species (sheep or goat), sex and age of every sampled animal were recorded.

### 2.2. Detection Method

The serum samples were tested for IgG antibodies against the MSP5 protein of *Anaplasma* spp. [50] by using a cELISA (Anaplasma Antibody Test Kit, cELISA v2, VMRD, Inc., Pullman, WA, USA). The assay was performed in accordance with the manufacturer’s instructions and is approved for the detection of antibodies against *A. marginale*, *A. ovis* and *A. centrale* in cattle. Results with an inhibition of ≥30% were specified as positive. This cELISA has already been successfully applied to ovine and caprine sera from areas where *A. ovis* and *A. phagocytophilum* are present [37,51].

### 2.3. Risk Analysis

Information about farm management and flock health were available from a recently performed Q fever study [48]. The standardised questionnaire consisted of questions concerning: (1) general farm indicators, (2) information on livestock kept on the farm, (3) the husbandry system, (4) flock history, and (5) last lambing season [48].

### 2.4. Statistical Analysis

#### 2.4.1. Seroprevalence

**Figure 2:**
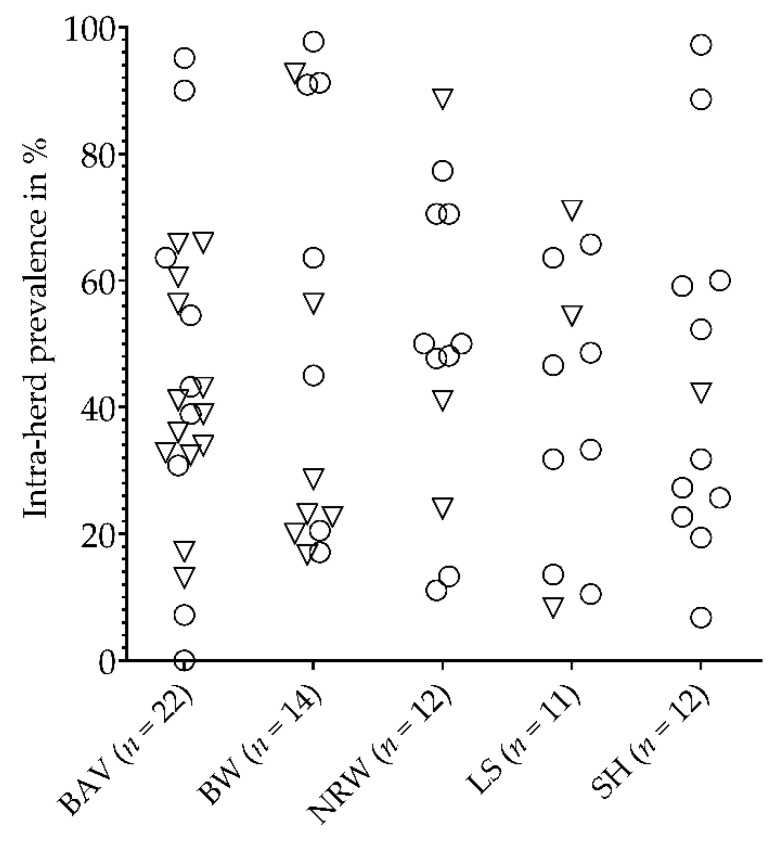
Intra-flock prevalences of antibodies to *Anaplasma* spp. of 71 small ruminant flocks from five German federal states: BAV = Bavaria, BW = Baden-Wuerttemberg, NRW = North Rhine-Westphalia, LS = Lower Saxony, SH = Schleswig-Holstein. ○ = pure sheep flock. ▽ = mixed sheep and goat flock.

The IFP of small ruminant flocks among the five federal states were tested for normal distribution followed by a Kruskal-Wallis test. To examine the distribution of the values of the individual animals between the federal states, the differences were calculated with an ANOVA. Subsequently, a test for least significant differences (Fisher’s Least Significant Difference (LSD) test) was carried out. In the southern federal states (BW, BAV) proportionally more goats were sampled than in the northern and western ones (SH, LS, NRW). Therefore, goats were excluded to avoid a distortion of the analysis and only the antibodies to *Anaplasma* spp. from the sheep are presented in Figure 2 (PROC LOGISTIC, SAS Institute, Inc., Cary, NC, USA).

#### 2.4.2. Risk Analysis

##### Correlation Analysis

First, all variables were verified in terms of their distinguishable content due to the large number of possible risk factors. A correlation analysis was carried out to support this step.

The following measures were determined and confirmed for correlation: Cramer’s V > 0.5 for qualitative variables, ANOVA (equal variances) or Kruskal-Wallis test (unequal variances) (*p* > 0.05) and coefficient of determination (R^2^ > 0.1) for qualitative and quantitative variables, and the Pearson correlation coefficient (>0.7) for quantitative variables. Either correlated variables were summarised, one of them removed for further analysis but considered in the subsequent interpretation of the results, or, if there was a moderate correlation, both variables were included in the model selection using an interaction term.

##### Risk Factor Analysis

A risk factor analysis was conducted to identify risk factors for exposure to *Anaplasma* spp. at animal and flock level. As almost all flocks tested *Anaplasma* spp. positive, the probability of acquiring an *Anaplasma* spp. seroprevalence of >20% was determined at flock level. The target variable was dichotomised (positive/negative). Furthermore, the geographical location was dichotomised (North = SH, LS, NRW; South = BAV, BW) to reduce the results’ distortion. Due to the different farm management systems in these two regions, the geographical location of the examined farms was considered as a confounder and therefore the model was stratified for the two regions.

For risk factor analysis at animal level, the farm was considered as a cluster variable. Therefore, an extended generalised linear model approach was chosen to take the hierarchical structure of the data into account. The parameters were estimated by using generalised estimating equations [52,53] (PROC GENMODE, SAS, Institute Inc.).

For the risk factors at flock level, univariable and multivariable logistic regression models were provided (PROC LOGISTIC, SAS, Institute Inc.). The model assumes the predictors (i.e., risk variables) $x_{1},\ldots ,\;x_{n}$ and a binary response variable Y (i.e., anaplasmosis <=/> 20%), with p = P (Y = 1). A linear relationship is assumed between the predictors and the logit function of Y = 1. Where ℓ is the logit function and b is the base of the logarithm:

$$\ell =\log_{b}\frac{p}{1-p}=\beta_{0}+\beta_{1}x_{1}+\ldots +\beta_{n}x_{n}$$

Odds ratio (OR), a 95% confidence interval (CI), Akaike Information Criterion (AIC) at flock level, or Quasi likelihood under the Independence Model Criterion (QIC) at animal level and *p* values were calculated for categorical and continuous variables. The Odds ratio was determined using:

$$\frac{p}{1-p}=b^{\beta_{0}+\beta_{1}x_{1}+\ldots +\beta_{n}x_{n}}$$

A variable was used for further analysis if it had a *p* value lower than 20% (*p* < 0.20) of the model [54]. Additionally, a distinctive OR < 0.75 or OR > 1.33 and a reasonable corresponding 95% CI (lCI > 0.001; uCI < 999.99) led to the variable being taken further into account. In rare cases, a variable took on the same value on all observed farms. As a result, it was impossible to calculate meaningful ORs and CIs and the corresponding variables were not considered for the multivariable models. These criteria allowed variables to be considered for further analysis if they did not have a *p* value lower than 20% but still had a distinctive OR. Hence, the multivariable model could be selected from the largest number of possible risk factors and the probability of wrongly removing influencing factors was minimised.

A forward selection was carried out with the variables that met the above-mentioned criteria. The variables which most improved the model fittings and whose addition achieved the best *p* values of the models were selected. The addition of the variables to the models was terminated either if all variables were included or if the addition of variables led to no further improvement of the model fittings and the *p* values. In the ultimate step, the final models were each examined for collinearity using the variance inflation factor.

## 3. Results

### 3.1. Occurrence of Anaplasma spp. in German Small Ruminant Flocks

In total, antibodies against *Anaplasma* spp. were detected in 70 out of 71 small ruminant flocks. The IFP ranged between 0% and 97.7%. However, the level of IFP did not differ in principle between the federal states (*p* > 0.05). There was only one sheep flock in BAV without antibodies to *Anaplasma* spp. (Figure 2).

**Figure 3:**
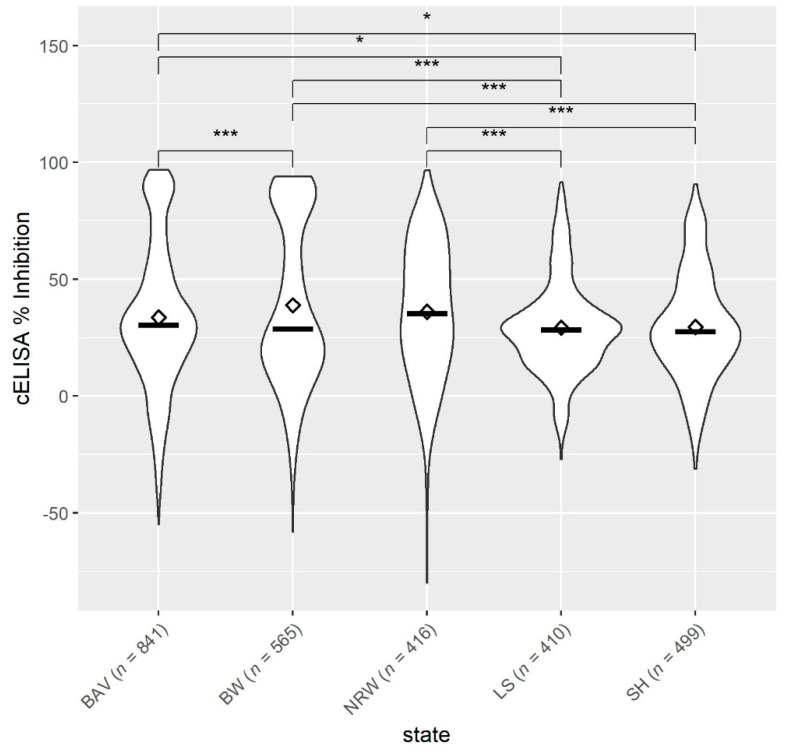
Mean ◊ and median—values of antibodies to *Anaplasma* spp. from sheep (*n* = 2731, 71 flocks) located in five German federal states: BAV = Bavaria, BW = Baden-Wuerttemberg, NRW = North Rhine-Westphalia, LS = Lower Saxony, SH = Schleswig-Holstein. * *p* < 0.05. *** *p* < 0.001.

Overall, the mean antibody levels against *Anaplasma* spp. in sheep from the northern federal states, SH and LS, were significantly lower compared to NRW, BW and BAV (Figure 3). Among the northern federal states, there was no difference in the sheep’s antibody response (*p* > 0.05). Moreover, the mean antibody levels were also not statistically different between BW vs. NRW and BAV vs. NRW (*p* > 0.05), but sheep from BAV had significantly lower *Anaplasma* spp. values than animals from BW.

### 3.2. Univariable Analysis

#### 3.2.1. Risk Factors at Animal Level for Exposure to *Anaplasma* spp.

Sheep had a 2.5 times higher risk acquiring an *Anaplasma* spp. infection than goats. Females had a 37% increased chance of being seropositive, but the likelihood of antibody detection in young animals (≤2 years) was reduced by one quarter (Supplementary Table S1).

#### 3.2.2. Risk Factors at Flock Level for Exposure to *Anaplasma* spp.

At flock level, only landscape conservation and cats had a significant *p*-value (Supplementary Table S2). Small ruminant flocks used for landscape conservation were four times more likely to have an IFP of above 20%. Farms with cats had an almost four-fold higher risk of having more than 20% IFP than farms without cats.

### 3.3. Multivariable Analysis

#### 3.3.1. Risk Factors at Animal Level for Exposure to *Anaplasma* spp.

The results of the multivariable analysis were in line with the univariable analysis and revealed no additional information (Table 1).

#### 3.3.2. Risk Factors at Flock Level for Exposure to *Anaplasma* spp.

The resulting multivariate model at flock level included four risk factors (Table 2) which were all significant except for contact to deer.

Small ruminant flocks which performed landscape conservation had about a five times higher risk of having more than 20% seropositive animals compared to flocks without this farming purpose. Observations of deer near the flock reduced the risk to less than one-sixth, while the presence of cats and dogs on the farms increased the probability of having an IFP above 20% 10-fold and 166-fold, respectively.

## 4. Discussion

Information about *Anaplasma* spp. in the German sheep and goat population is still scarce [4,8] despite *A. phagocytophilum* having been well described in horses [18,19], cattle [20,21], dogs [22,23], cats [24,25] and wild ruminants [9,55] from Germany.

### 4.1. Occurrence of Anaplasma spp. in German Small Ruminant Flocks

In the present study, antibodies to *Anaplasma* spp. were determined in almost all small ruminant flocks, indicating a high distribution of *Anaplasma* species across the country which is consistent with molecular findings in small ruminants in different parts of Germany [4,8,47]. The comparison of our serological results with findings from other studies is hampered due to the different study designs and methods to identify antibodies to *Anaplasma* spp. in small ruminants.

However, our results are similar to serological studies from Italy, Hungary and the US, which also detected a high seroprevalence in small ruminants by using the same cELISA. In Italy, antibodies against *Anaplasma* spp. were detected in 69.59% (*n* = 217) and 45.45% (*n* = 22) of the examined sheep and goats, respectively [56]. In a subsequent study, almost all sheep (98%) in a single flock (*n* = 200) from Italy had antibodies to *Anaplasma* species [37]. A high detection rate was also reported from five sheep flocks (*n* = 156) in Hungary, with 99.4% seropositive animals [2] and from a sheep flock (*n* = 357) from Idaho (USA) with an antibody response to *A. ovis* of 94.8% [5]. In contrast, the presence of antibodies to *Anaplasma* species varied from 0% to 43.3% among eight sheep flocks in California and Oregon (USA) determined by cELISA and indirect immunofluorescence assay [6]. The mean antibody levels in sheep from the northern federal states (SH, LS) were significantly lower compared to the levels from sheep in the West (NRW) and the South (BW, BAV) of Germany. This might indicate a different exposure to ticks and to *Anaplasma* species of sheep and goats in different parts of the country. Despite *I. ricinus* being widely distributed across Germany [57], sheep from LS and SH may have less contact to ticks. In these federal states, sheep play an essential role for coastal protection by grazing on dykes. These areas usually have a low vegetation which is less preferred by ticks [58,59]. In contrast, sheep flocks in the South are widely used for landscape conservation and grazing on forest edges and scrubs representing the natural habitat of ticks [58,59]. This hypothesis is supported by our findings from the multivariable risk factor analysis. Small ruminant flocks which performed landscape conservation had a five times higher likelihood of having an IFP above 20%. Moreover, the focal dissemination of *Dermacentor* spp. in NRW, BW and BAV [57,60] as a potential vector for *A. ovis* and the recent detection of this pathogen in BAV [4] might also influence the higher antibody response in small ruminants from these areas compared to animals from Northern Germany.

### 4.2. Risk Factor Analysis on Animal Level

The likelihood of sheep testing positive for antibodies to *Anaplasma* spp. was 2.5 times higher than for goats. This corresponds to observations from other serological studies in which the percentage of seropositive animals was higher in sheep than in goats [56,61]. Sheep and goats responded differently to *A. phagocytophilum* regarding the severity of clinical disease and haematological changes [26]. These might also result in differences in production and longevity of antibodies against *Anaplasma* species. Additionally, it might be possible that ticks are attracted to a varying extent by sheep and goats due to their different odour. Some tick species might prefer individual mammal species and are attracted by the specific host’s odour, but *I. ricinus* seems not to have any preferences [62,63]. However, the conducted studies did not include goats as targeted species.

Due to the lack of *Anaplasma* risk factor analyses for sheep or goats in Europe and North America, we will discuss our outcomes in a broader context. Older animals (>2 years) and female small ruminants had a higher risk of becoming exposed to *Anaplasma* spp. in the current study. In general, older animals might be exposed to ticks for a longer period of time, which increases the chance of being exposed to *Anaplasma* species. This is supported by findings from *A. ovis* DNA positive goats in France [64]. Animals older than three years tested positive significantly more often. However, age was not related to *Anaplasma* spp. antibody levels in sheep from Hungary [2]. Furthermore, antibodies against *A. phagocytophilum* were detected age-independent in horses from the Czech Republic [65]. These conflicting results might occur due to differences in *Anaplasma* species, environmental conditions and animal husbandry. Similar to our findings, mares were more likely to be *A. phagocytophilum* seropositive than stallions [65]. Generally, male breeding animals are managed differently compared to females; for instance, males graze on a separate pasture or are kept indoors. The latter especially reduces the likelihood of tick infestation and thus the risk of *Anaplasma* spp. exposure.

### 4.3. Risk Factor Analysis on Flock Level

The presence of cats on farms significantly increased the risk of *Anaplasma* spp. exposure in small ruminants. In Germany, there is evidence of *A. phagocytophilum* infections in cats. A seroprevalence of 16.7% (*n* = 326) was detected in cats from LS and BAV [24] and a proportion of 23% *A. phagocytophilum* seropositive cats (*n* = 956) were reported from a nationwide study [25]. However, both studies examined sera from pet cats which might have less access to tick habitats compared to barn cats. Therefore, the infection rate with *A. phagocytophilum* might be higher in the barn cat population. This must be validated in the future. Like cats, dogs were also identified as a risk factor for small ruminant flocks having an IFP greater than 20%. Their presence on farms increased the risk 166-fold. In total, only three farms (*n* = 71) had no dogs and from this group, one flock had an IFP of >20%. This small sample size might have distorted our findings and probably overestimates the risks of dogs. Nevertheless, canine granulocytic anaplasmosis has been regularly diagnosed in Germany and an *A. phagocytophilum* seroprevalence between 43.2% and 50.1% was reported within the dog population [22,66]. Finally, dogs and cats are hosts for *A. phagocytophilum* but their epidemiological role for *Anaplasma* spp. infections in small ruminants remains unclear.

The deer population is considered to be a reservoir for *A. phagocytophilum* and *A. ovis* in several countries [3,7,8,9,10,11,67,68]. Molecular investigations determined high infection rates of *A. phagocytophilum* in roe deer (*Capreolus capreolus*, 98.9%), red deer (*Cervus elaphus*, 100%), sika deer (*Cervus nippon*, 86.4%) and fallow deer (*Dama dama*, 72.1%) in Germany [9,46,69]. In the present study, the farmers were not asked to distinguish between deer species when these animals were in the vicinity of their small ruminant flocks. Our findings indicate that deer did not increase the likelihood of exposure to *Anaplasma* spp. in small ruminants. The roe deer population seems to have its specific *A. phagocytophilum* variants and gene clusters, which are rarely found in sheep [4,8,70]. In contrast, red deer and sheep might share some *A. phagocytophilum* variants/gene clusters [4,8,70,71]. Therefore, the interaction of *Anaplasma* spp. between sheep and different deer species needs further clarification.

The observed tick infestation by the farmers and the ectoparasitic treatment had no effect on the seropositivity of the sheep and goats, which is in line with findings from a previous study on tick-borne encephalitis virus infections in small ruminants [72]. Although a significant reduction in tick infestation was achieved after the prophylactic treatment of lambs with flumethrin, the seroprevalence was not reduced through the treatment [73]. Consequently, other preventive measures are necessary to protect small ruminants from exposure to *Anaplasma* species.

Species differentiation by cELISA is not possible and is a limitation of our study. Hence, molecular investigations are crucial to distinguish between *A. phagocytophilum*, *A. ovis* and possible other *Anaplasma* species. The recent detection of *A. ovis* for the first time in a German sheep flock underlines the necessity of species identification [4]. Furthermore, *A. phagocytophilum* is widely distributed within the German small ruminant population, knowledge on different genetic variants is scarce but the limited numbers of studies indicate a large diversity of *A. phagocytophilum* variants [4,8,47]. Therefore, molecular analyses of *Anaplasma* spp. isolates from sheep and goats may reveal new insights into the epidemiological situation and the possible clinical impact of different *Anaplasma* variants on sheep and goats in Germany.

## 5. Conclusions

The present study generated important information about the dissemination of *Anaplasma* spp. infection within the small ruminant population in Germany and contributes to a better understanding of the complex epidemiology of anaplasmosis in sheep and goats. Almost all small ruminant flocks tested seropositive for *Anaplasma* spp. antibodies. Moreover, due to the wide dissemination of *Anaplasma* spp. in the German small ruminant population, an infection with these pathogens is probably completely underdiagnosed. Thus, in cases of unspecific clinical signs like high fever, anorexia, anaemia, dullness and poor growth rate in sheep and goats, veterinarians should rule out an infection with *Anaplasma* species.

## Figures and Tables

**Table 1:** Multivariable risk factor model at animal level for exposure to *Anaplasma* spp.

Variable	Category	Odds Ratio (OR)	95% Confidence Interval	*p*-Value	Quasilikelihood under the Independence Model Criterion (QIC)
Species	Goat	2.525	1.443–4.417	0.001	4342.598
Sex	Male	1.378	1.029–1.846	0.032	
Age	>2 years	0.739	0.970–0.970	0.029	

**Table 2:** Multivariate risk factor model at flock level for the risk of having an *Anaplasma* spp. intra-flock seroprevalence of above 20%.

Variable	Category	Odds Ratio (OR)	95% Confidence Interval	C- *p*-Value	LR- *p*-Value	Akaike Information Criterion (AIC)
Landscape conservation	No	5.348	1.026–7.877	0.047	0.0002	49.614
Deer	No	0.149	0.015–1.461	0.102		
Cats	No	10.731	1.681–68.514	0.012		
Dogs	No	166.328	3.606–>999.999	0.009		

## Data Availability

The data are available on request from the corresponding author.

## Supplementary Materials

The following are available online at https://www.mdpi.com/article/10.3390/ani11102793/s1, Table S1: Univariable risk analysis at animal level for sheep and goats to be *Anaplasma* spp. seropositive. Table S2: Univariable risk analysis at flock level for the risk of having an *Anaplasma* spp. intra-flock seroprevalence of above 20%.
