# Supplementary material for: Seroprevalence and Risk Factors of Anaplasma spp. in German Small Ruminant Flocks
Source: Animals (Basel). 2021 Sep 25;11(10):2793. doi: 10.3390/ani11102793 (PMC8532635; doi:10.3390/ani11102793)
Supplement: Supplementary file 1 [file animals-11-02793-s001.zip › animals-1380762-supplementary.pdf]

Supplementary Materials

Table S1. Univariable risk analysis at animal level for exposure to *Anaplasma* spp.

Variable	Category	Apparent Prevalence of Positive Animals/ Animals Total (%)	Odds Ratio (OR)	95% Confidence Interval	*p*-Value	Quasilikelihood under the Independence Model Criterion (QIC)
Species	Goat	127/447	2.482	1.418–4.344	0.002	4355.870
	Sheep	1336/2731				
Sex	Male	151/399	1.373	1.027–1.834	0.032	4401.188
	Female	1312/2779				
Age	>2 years	1294/2768	0.753	0.578–0.980	0.035	4408.810
	≤2 years	169/410				

Table S2. Univariable risk analysis at flock level for the risk of having an *Anaplasma* spp. intra-flock seroprevalence of above 20%.

Variable	Category	Apparent Prevalence of Positive Farms/Farms Total (%)	Odds Ratio (OR)	95% Confidence Interval	C-*p*-Value	LR-*p*-Value	Akaike Information Criterion (AIC)
Husbandry system	Paddock	44/56	1.811	0.344–9.539	0.484	0.465	64.784
	No Paddock	13/15					
Landscape conservation	No	15/23	4.057	1.138–14.459	0.031	0.028	60.489
	Yes	42/48					
Location	Forest edge	44/53	0.531	0.150–1.886	0.328	0.336	64.391
	Others	13/18					
Infestation with ticks	No	12/15	1.012	0.237–4.324	0.987	0.987	65.317
	Yes	45/56					
Ectoparasitic treatment	No	21/27	1.282	0.396–4.145	0.678	0.678	65.147
	Yes	36/44					
Injections	No injections	8/9	>999.999	<0.001 >999.999	0.993	0.375	65.355
	No needle change	3/3					
	No injections	8/9	0.446	0.051–3.865	0.464		
	With needle change	46/59					
Cattle	No	36/46	1.446	0.409–5.112	0.567	0.560	64.978

	Yes	21/25					
Swine	No	51/64	1.510	0.171–13.358	0.711	0.699	65.168
	Yes	6/7					
Poultry	No	29/37	1.277	0.397–4.107	0.682	0.681	65.148
	Yes	28/34					
Wild birds	No	5/6	0.798	0.087–7.303	0.841	0.838	65.276
	Yes	52/65					
Deer	No	12/13	0.295	0.036–2.451	0.258	0.193	63.625
	Yes	43/58					
Dogs	No	1/3	8.705	0.761–99.505	0.082	0.073	62.102
	Yes	56/68					
Cats	No	22/32	3.893	1.094–13.850	0.036	0.028	60.456
	Yes	35/39					
Rodents	No	7/10	1.906	0.436–8.323	0.391	0.407	64.629
	Yes	50/61					
Lambing behaviour	Seasonal	38/47	0.844	0.201–3.538	0.817	0.817	65.264
	Aseasonal	19/24					
Abortion	No increased abortion rates	51/65	>999.999	<0.001 >999.999	0.994	0.129	62.561
	Increased abortion rates	5/5					
Pneumonia in lambs	No	48/61	2.458	0.285–21.209	0.413	0.368	64.506
	Yes	9/10					
Diseases in dams	No	48/61	2.412	0.280–20.751	0.423	0.378	64.541
	Yes	9/10					
